# Electrochemical Sensors for the Detection of Lead and Other Toxic Heavy Metals: The Next Generation of Personal Exposure Biomonitors

**DOI:** 10.1289/ehp.10190

**Published:** 2007-09-21

**Authors:** Wassana Yantasee, Yuehe Lin, Kitiya Hongsirikarn, Glen E. Fryxell, Raymond Addleman, Charles Timchalk

**Affiliations:** 1 Pacific Northwest National Laboratory, Richland, Washington, USA; 2 Department of Chemical Engineering, Chulalongkorn University, Bangkok, Thailand

**Keywords:** biomonitoring, dosimetry technology, electrochemical sensors, exposure assessment, lead (Pb)

## Abstract

To support the development and implementation of biological monitoring programs, we need quantitative technologies for measuring xenobiotic exposure. Microanalytical based sensors that work with complex biomatrices such as blood, urine, or saliva are being developed and validated and will improve our ability to make definitive associations between chemical exposures and disease. Among toxic metals, lead continues to be one of the most problematic. Despite considerable efforts to identify and eliminate Pb exposure sources, this metal remains a significant health concern, particularly for young children. Ongoing research focuses on the development of portable metal analyzers that have many advantages over current available technologies, thus potentially representing the next generation of toxic metal analyzers. In this article, we highlight the development and validation of two classes of metal analyzers for the voltammetric detection of Pb, including: *a*) an analyzer based on flow injection analysis and anodic stripping voltammetry at a mercury-film electrode, and *b*) Hg-free metal analyzers employing adsorptive stripping voltammetry and novel nanostructure materials that include the self-assembled monolayers on mesoporous supports and carbon nanotubes. These sensors have been optimized to detect Pb in urine, blood, and saliva as accurately as the state-of-the-art inductively coupled plasma-mass spectrometry with high reproducibility, and sensitivity allows. These improved and portable analytical sensor platforms will facilitate our ability to conduct biological monitoring programs to understand the relationship between chemical exposure assessment and disease outcomes.

Biological monitoring (biomonitoring) can integrate total exposure to a chemical from all potential sources (i.e., air, soil, water, and food residues) and exposure routes (i.e., inhalation, oral, and dermal), thereby offering one of the best approaches for assessing human dosimetry. Hence, the primary strength of biomonitoring is the ability to measure an internal dose for a given chemical (Gil and Pla 2001) that can then be used to determine risk from exposure to these agents (Christensen 1995; Friberg and Elinder 1993). Weis et al. (2005) recently noted that current epidemiology study designs are seriously hampered because of their inability to make definitive associations between chemical exposures and disease. However, the authors suggested that the integration of well-designed biomonitoring components within the framework of an epidemiologic evaluation offers the greatest potential to positively impact the public health strategy as it relates to the identification of important environmental health issues.

The greatest impediment to conducting epidemiology studies is the lack of accurate and quantitative technologies for measuring exposure at the point of human contact and within the organism (Weis et al. 2005). To address these limitations, microanalytical-based sensors are needed that are field deployable and can be used to both identify and quantify a broad range of biomarkers associated with chemical exposures (Timchalk et al. 2004; Weis et al. 2005). As reviewed by Weis et al. (2005), these sensor platforms offer great promise because they have the potential to provide a rapid, accurate, and quantitative detection of exposure at the level of the individual. Therefore, they effectively couple environmental and personal exposure assessments to a broad range of chemical agents in a quantifiable and meaningful way that will enhance the design and interpretation of epidemiology studies.

Among toxic heavy metals, lead continues to be one of the most problematic. Despite considerable efforts to identify and eliminate Pb exposure sources, this metal still remains a significant health concern [Banks et al. 1997; Grigg 2004; Juberg et al. 1997; U.S. Centers for Disease Control and Prevention (CDC) 2000] and the guidelines for potential health effects are linked directly to measured blood Pb levels (Jakubowski and Trzcinka-Ochocka 2005). Since the 1960s the CDC has gradually reduced the blood Pb intervention level from 60 μg/dL to the current intervention level of 10 μg/dL (established in 1991) for children. However, Gilbert and Weiss (2006) suggest that further lowering of the action level to 2 μg/dL is warranted because of evidence suggesting intellectual impairment in children with blood Pb levels ≤ 10 μg/dL (Canfield et al. 2003). It is interesting to note that the CDC Surveillance Summaries (CDC 2003) published from 1997 to 2001 suggest that children’s blood Pb levels in the United States continue to decline; however, thousands of children still have elevated levels (≥ 10 μg/dL). In addition, Pb exposure in children from many developing industrial countries far exceeds current U.S. guidelines such that Pb is still a significant world health concern (Blaha et al. 1996; Cikrt et al. 1997; Manay et al. 1999; Romieu et al. 1997). In this context, it is reasonable to anticipate that any further lowering of the blood Pb action levels will substantially increase the number of children at or above the intervention level.

Biomonitoring of Pb in humans represents an individual’s current body burden, which is a function of recent and past exposures (Barbosa et al. 2005). Because Pb is sequestered in bone and has a relatively long residence time, steady-state concentrations of Pb in whole blood are currently used to determine total Pb body burden (Beck 1992). Although blood Pb remains the biomonitoring method of choice, a clear statistical correlation between blood Pb and adverse health outcomes has not always been strong (Barbosa et al. 2005). In this regard, Hu et al. (1998) suggested that blood Pb may not be a sufficiently sensitive biomarker, particularly in light of the *in vivo* dynamics of Pb tissue redistribution. Consequently, there is substantial interest in identifying alternative Pb biomarker matrices such as plasma/serum, saliva, bone, teeth, feces, and urine (Barbosa et al. 2005), which might provide a better correlation with adverse health outcomes.

In this article we provide an overview of ongoing research focused on the development and validation of differing classes of portable electrochemical-based metal analyzers that have the potential to become the next generation of toxic metal analyzers. An important component of the overall research effort has been the optimization of the sensor systems to work with complex biomatrices such as blood, urine, or saliva. Validation of these sensor platforms for use in biomonitoring is particularly important in developing a personalized exposure assessment strategy, as suggested by Weis et al. (2005), that may improve our ability to make definitive associations between chemical exposures and disease.

## Pharmacokinetics of Pb

Lead is a bone-seeking element that has a very long residence time (i.e., months to years) in the human body. In children (most at-risk population) the oral route of exposure predominates and Pb absorption within the gastrointestinal tract is as high as 40–50% (Bellinger 2004; Erickson and Thompson 2005). Within the blood compartment, Pb is rapidly partitioned between red blood cells (RBCs) and plasma, with RBCs accounting for 95% of the blood Pb burden (Marcus 1985; Mobarak and P’an 1984). Pb is then redistributed to the bone (~ 70%) and soft tissues and is excreted slowly with its biological half-life estimated at 10 years (Erickson and Thompson 2005; Heath et al. 2003).

Although blood measurements represent the most common strategy for Pb biomonitoring because of the strong association between RBCs and Pb, several studies suggest that alternative matrices such as plasma, saliva, and urine may be useful (Timchalk et al. 2006). Heavy metals such as Pb are excreted into the feces via the bile or from the blood into the urine. Of these two excretion pathways, urine is the preferred matrix for biomonitoring, as it represents only absorbed Pb, whereas fecal Pb comprises both unabsorbed as well as biliary excreted Pb (Barbosa et al. 2005). The rate of urinary Pb excretion is reported to be directly proportional to the plasma Pb concentration; hence, urinary Pb reflects that fraction of Pb that has cleared from the plasma via the kidney and excreted in urine (Barbosa et al. 2005; O’Flaherty 1993, 1998). However, the application of Pb urinary biomonitoring has been primarily limited to longer-term occupational biomonitoring programs and the evaluation of the efficacy of chelation therapy (Barbosa et al. 2005). Nonetheless, urinary Pb biomonitoring does offer an alternative noninvasive approach.

Although blood Pb measurement is still considered the most reliable indicator of recent Pb exposure, it has also been suggested that if reliable plasma Pb measurements can be obtained, these measurements may offer a better correlation with observed toxicity (Barbosa et al. 2005). In this context, correlations between labile Pb concentrations in plasma with either saliva or urine suggest that these matrices may offer an alternative to current invasive biomonitoring procedures.

## Challenges Associated with Sensor Development

Biomonitoring of Pb in individuals presently relies on collection of biological samples for subsequent laboratory analysis by means of standard spectroscopic techniques such as atomic absorption spectrometry (AAS) and inductively coupled plasma–mass spectrometry (ICP-MS). These analytical methods are generally conducted in centralized laboratories and require significant labor and analytical resources, potentially resulting in substantial delays in obtaining results. Desirable characteristics of a portable metal analyzer include specificity for target metal ions, enhanced measurement frequency and precision, robustness, inexpensive to fabricate and operate, ability to be automated, and minimal regeneration of sensors. Electrochemical detection based on stripping voltammetry appears to be a promising technique that meets those needs (Lin et al. 1999; Wang 1994; Wang et al. 1993a). Its high detection sensitivity is due to the combination of the built-in preconcentration step with powerful voltammetric techniques that generate an extremely favorable signal-to-noise ratio (S/N).

For biomonitoring of toxic metals, the complexity of the biological matrices such as urine, blood, and saliva often prevents successful use of electrochemical sensors. Over 95% of Pb became bound to saliva proteins within 2 min of spiking Pb into the sample (Yantasee et al. 2005c). The binding of target metals to proteins and macromolecules in the biological matrices can result in a low voltammetric response to known concentrations of the metals (Timchalk et al. 2001; Wang 1982). Proteins also contribute to electrode fouling, which results in significant signal reduction and shortening of the electrode life time. To overcome these issues, researchers have used techniques such as application of ultrasound wave during the depositions of metal ions together with coating mercury-film electrodes with polymers [e.g., nafion (Kruusma et al. 2004; West et al. 2002)], yet large dilution of the samples is still required. Another approach involves using internal standards [e.g., indium (Liu et al. 1997) and thallium (Yang et al. 2005)] to compensate for the biological matrix effect on the metal analysis. However, this often increases the measurement complexity. Other approaches involve enzyme-based biosensors [e.g., urease (Rodriguez et al. 2004), oxidase (Gayet et al. 1993), and dehydrogenase (Fennouh et al. 1998)], which detect metal ions (e.g., Hg, Pb, zinc, copper, cadmium, silver) by relying on their inhibition of enzymatic reactions. However, the biosensors have modest sensitivity (e.g., in high parts per billion or parts per million levels). To become commercially viable, biosensor design must overcome issues including detection interference from many components in the sample matrices, limited shelf-life of the biorecognition components, relatively high development costs, and the complexity of the field-deployable assay formats (Rogers and Mascini 2006)

## Sensor Development Strategies

In our laboratory, releasing Pb from the proteins and minimizing electrode fouling in urine (Yantasee et al. 2007), blood (Yantasee et al. 2007), and saliva (Yantasee et al. 2005c) have been accomplished with the combination of appropriate sample pretreatment and the optimal configurations and operation of the sensors. The approaches are discussed herein.

**Figure 1:**
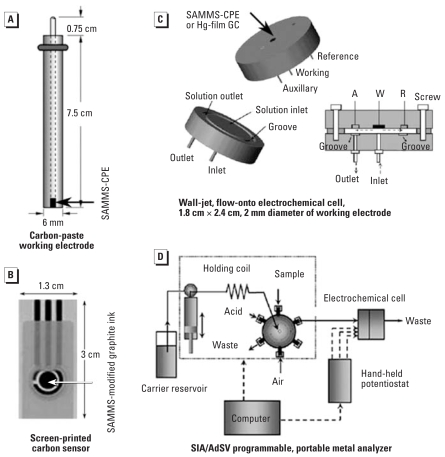
Various electrochemical sensors for metal detection: (*A*) carbon paste working electrode for batch detection, (*B*) screen-printed electrode, (*C*) electrochemical cell, and (*D*) schematic of a portable metal analyzer [10.6 in (length) × 9.7 in (width) × 6.9 in (diameter)]. Abbreviations: A, auxillary; R, reference; W, working. Reproduced from Yantasee et al. (2005b), with permission of Elsevier.

For the voltammetric detection of Pb, two classes of metal analyzers have been developed in our laboratory: *a*) a metal analyzer based on flow injection analysis (FIA) and anodic stripping voltammetry (ASV) at a Hg-film electrode (Yantasee et al. 2005c, 2007) and *b*) Hg-free metal analyzers employing novel nanostructure materials, which include the self-assembled monolayers on mesoporous supports (SAMMS) (Yantasee et al. 2005b) and composite of SAMMS and carbon nanotubes (CNTs). Figure 1 presents the sensor platforms being investigated, including *a*) carbon paste electrode (CPE) for batch measurements, *b*) disposable screen-printed electrode (SPE) for low-cost and field-screening measurements, and *c*) electrochemical cell that is the major component of *d*) the programmable portable metal analyzer. Their uses are reviewed in the sections that follow.

## Portable Metal Analyzer Based on FIA/ASV at a Hg-Film Electrode

To minimize electrode fouling by proteins, we hypothesize that the turbulent flowing of the samples that are appropriately pretreated onto the electrode surface can prevent the adsorption of organic species on the sensor surface. For that purpose, FIA has been employed in conjunction with ASV technique for successful measurements of Pb in saliva, blood, and urine. The schematic of the microelectrochemical cell, based on a wall-jet (flow-onto) design, is presented in Figure 1C. The FIA system is similar to the sequential injection analysis (SIA) system shown in Figure 1D except that a computer-controlled micropump delivered the carrier passing the two-way injection valve where an exact volume of the sample was then injected. Typical operating conditions of this metal analyzer and the sample preparation protocol are summarized in Table 1.

**Figure 2:**
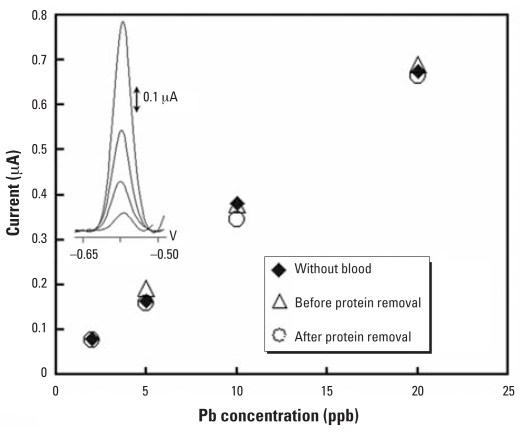
Pb signals as a function of Pb concentrations in solution without blood and with blood samples prepared by spiking Pb before and after proteins are removed; inset shows the corresponding voltammograms of Pb in 10-vol% blood sample. Reproduced from Yantasee et al. (2007), with permission of Springer.

Figure 2 shows that Pb spiked either before or after protein removal from the blood by ultrafiltration yielded similar responses, which were also similar to Pb samples containing no blood. This suggests that the pretreatment protocol as in Table 1 was effective in freeing Pb from the proteins into the ultrafiltrate. In addition to releasing Pb from the proteins, the acid (0.5 M HCl) also helped minimize the adsorption of residual organic species (< 5,000 Da) on the electrode surface.

**Figure 3:**
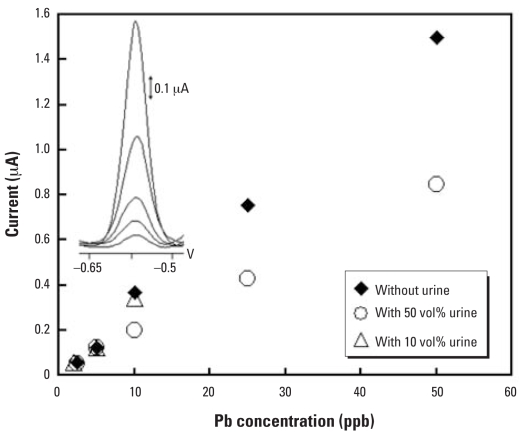
Pb signals as a function of Pb concentrations in samples with 0, 10, and 50 vol% of urine; inset shows the corresponding Pb voltammograms in 50%-urine samples. Reproduced from Yantasee et al. (2007), with permission of Springer.

In addition to the turbulent flow of the samples over the electrode surface and the acidity of the samples, cleaning of the electrode at a positive potential (0.6 V for 90 sec) in the carrier solution also removed the residual proteins along with Hg film and Pb, hence minimizing fouling of the electrode. As a result, the microanalyzer yielded excellent reproducibility (in terms of relative standard deviation; %RSD) for Pb measurements in both blood (Figure 2) and urine samples (Figure 3). Specifically, in samples containing 10 vol% of blood, the %RSD of 10 consecutive measurements of 10 ppb Pb was 2.4 (Table 2), which was better than the 5% reported for the ICP-MS blood Pb analysis (Schutz et al. 1996). There was also no matrix effect for the analysis of the samples containing 50% saliva, 10% urine, and 10% blood, thus the calibration curve can be constructed in pure medium and used to calculate Pb from any sample type and donor. Although the matrix effect was apparent in samples containing 50% urine, the %RSD of 7 consecutive measurements of 10 ppb Pb was 4.9 (Table 2), suggesting that there was no electrode fouling at this high urine content. Other figures of merits [linear calibration parameters and lower detection limits (LDL)] of FIA/ASV at Hg-film electrodes are also summarized in Table 2 along with those from other types of metal analyzers that are discussed in the following section.

The linear calibration ranges measured from this metal analyzer as shown in Table 2 are highly relevant to the biomonitoring of Pb in individuals resulting from occupational and environmental exposures. Table 3 summarizes the Pb concentrations measured by ICP-MS and the microanalyzer in saliva, urine, and blood that were collected from rats and were spiked *in vitro* with Pb. The average values and errors were highly comparable between both methods, suggesting that the microanalyzer can be used effectively for on-site biomonitoring of Pb in lieu of the in-laboratory ICP-MS.

## Measurement of Pb, Cd, and Cu with FIA/ASV at a Hg-Film Electrode

**Figure 4:**
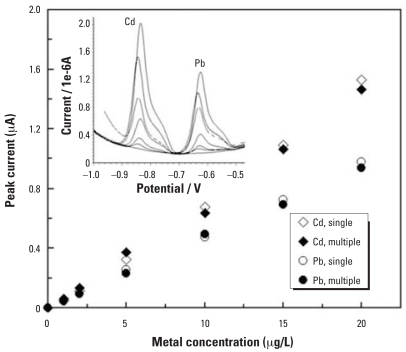
Voltammetric responses of Cd and Pb at the microanalyzer, measured in single component Pb and Cd solutions and a multicomponent Pb/Cd solution. Operating conditions are described in Table 1; inset shows the corresponding voltammograms.

**Figure 5:**
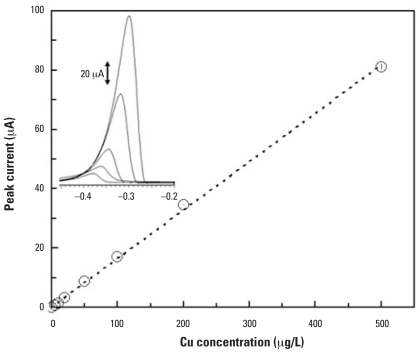
Voltammetric responses of Cu at the microanalyzer. Operating conditions are described in Table 1; inset shows the voltammograms of 5, 10, 20, 50, and 100 μg/L of Cu. *y* = 0.16*x; R*^2^ = 1.00

The microanalyzer has versatility in detecting other metals in addition to Pb. Figure 4 shows linear calibration curves of Cd and Pb measured in single component Pb and Cd solutions and a multicomponent Pb–Cd solution. The Pb and Cd signals were preserved when they were detected together compared with when they were detected separately. This suggests that the microanalyzer could simultaneously detect both Cd and Pb without losing the detection sensitivity. With the same analyzer, Cu detection has also been accomplished as shown in Figure 5. Note that the calibration curve was extended to at least 500 μg/L of Cu to be relevant to blood Cu concentrations, which are normally in parts per million levels. The large linear range from 0 to 500 μg/L of Cu suggests that the sensor surface is not easily saturated by metal ion accumulation.

## Hg-Free, SAMMS-Based Metal Analyzers

**Figure 6:**
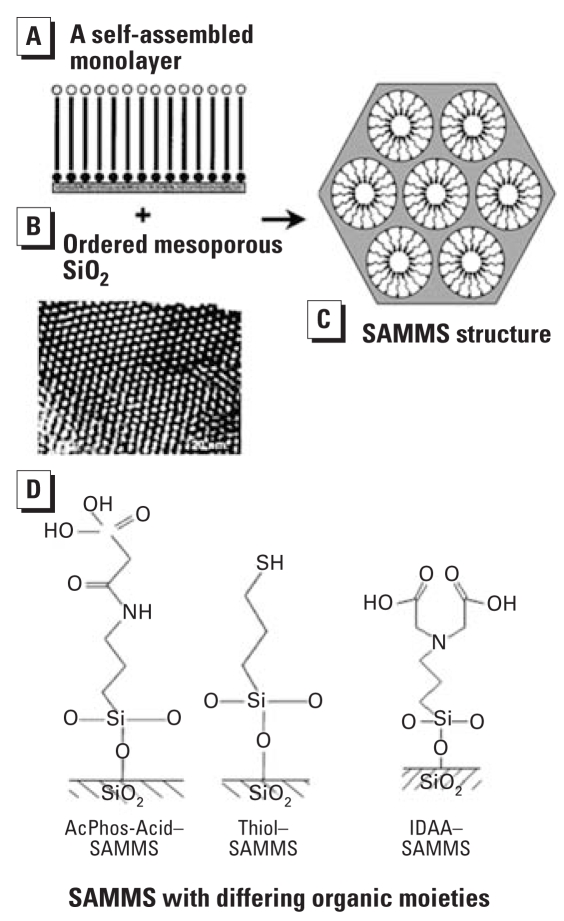
A hybrid of (*A*) self-assembled monolayer and (*B*) ordered mesoporous silica resulting in (*C*) SAMMS structure with (*D*) three differing organic moieties as the monolayers. SiO_2_, silicon dioxide.

Unlike conventional Hg-based sensors, the metal microanalyzer mentioned above required extremely small quantity of Hg (e.g., 1 μg of Hg per measurement). However, having considered the toxicity of Hg which may lead to future regulation of Hg use in sensors, we have developed new classes of metal sensors that are Hg-free, yet retain the sensitivity of the Hg-based sensors. Instead of using Hg to preconcentrate Pb, highly sorbent materials developed at Pacific Northwest National Laboratory (PNNL), the self-assembled monolayer on mesoporous supports (SAMMS), have been used. SAMMS materials are hybrid of ordered mesoporous silica (Figures 6A–C) and organic moieties (Figure 6D), which have been tailored to be specific for the sorption of metal ions (e.g., soft metal cations, anions, lanthanides, and actinides). SAMMS possess all the desired characteristics for metal ion preconcentration at an electrode surface, including *a*) high selectivity for target metals, *b*) high loading capacity, *c*) fast sorption kinetics, *d*) excellent stability because of the covalent bonding between organic molecules and silica substrate and cross-linking of the silane linkages, and *e*) the ability to be regenerated. Because of their high binding affinity for Pb, three SAMMS materials functionalized with thiol (SH), acetamide phosphonic acid (AcPhos), and iminodiacetic acid (IDAA) (Figure 6D) have been investigated in our laboratory for the preconcentration of Pb.

To construct a working electrode, SAMMS have been used in the mixture of SAMMS–carbon paste, SAMMS–graphite ink, or SAMMS–CNT paste. These working electrodes have then been used in batch measurements (Figure 1A), a portable metal analyzer based on sequential injection analysis/adsorptive stripping voltammetric analysis [SIA/adsorptive stripping voltammetry (AdSV)] (Figure 1D), and in disposable sensors for rapid screening of Pb (Figure 1B).

## SAMMS–Carbon Paste Electrodes

SAMMS–carbon paste electrodes (CPEs) were typically constructed by mixing SAMMS powder with carbon paste (graphite powder plus mineral oil) to obtain 5–10 wt% SAMMS consistency. AdSV at SAMMS–CPEs involved a two-step process: a preconcentration step at the open circuit, followed by a medium exchange to a pure electrolyte solution for the voltammetric quantification. During the pre-concentration step (2–5 min in a stirred sample), Pb(II) was accumulated on SAMMS, which was immobilized on the carbon paste by complexation chemistry.This technique allows the preconcentration of metal ions without electrolyte, applied potential, or degassing of the solution. Because SAMMS is an electronic insulator, to be detected sensitively, Pb bound to SAMMS must be first released to the conductive matrix. Thus, in the electrolysis step, accumulated Pb^2+^ was desorbed in an acidic medium (0.3–0.5 M HCl or HNO_3_) and simultaneously electrolyzed by applying a negative potential (–0.9 V) for about 60 sec to convert metal ion (Pb^2+^) to elemental metal Pb^0^. In the detection step, the elemental metal Pb^0^ was subsequently stripped by square-wave stripping voltammetry (SWV) technique (e.g., scanning from –0.8 to –0.3 V) to yield a Pb anodic peak at about –0.4 to –0.6 V. After the stripping step, cleaning often was not required, as in 0.3–0.5 M acid, Pb that was stripped out did not readsorb onto the SAMMS.

### Batch measurements

For batch Pb measurements, SH–SAMMS–CPE was packed into one end of a rod electrode (Figure 1A). The synthesis of SH–SAMMS has been described elsewhere (Feng et al. 1997). SH–SAMMS has a very high affinity for Hg, Ag, gold, Cu, Cd, and Pb. When SH–SAMMS sensors are used to evaluate Pb in complex groundwater, the sensors can readily detect Pb without significant sample pretreatment other than adjusting the sample pH to 6.5. The detection limits, linear range, and %RSD of Pb in groundwater are summarized in Table 2. The sensor has a detection limit of 2 ppb Pb, which is better than the U.S. Environmental Protection Agency (EPA) action level for Pb (15 μg/L) in public drinking water supplies (EPA 2007).

### Automated metal analyzer based on SIA/AdSV

To develop the next-generation prototype analyzer, we have integrated a SIA with the SAMMS–CPE employing AdSV. Figure 1D illustrates the SIA system that included an electrochemical flow cell (Figure 1C). Inside the cell, the working electrode consisted of AcPhos–SAMMS/carbon paste material. The synthesis protocol and characterization of AcPhos–SAMMS were published elsewhere (Birnbaum et al. 2002; Yantasee et al. 2003). All measurement steps were automated, which allows for the interchanging of fluids automatically and without interrupting the operation. After a 2-min pre-concentration period, the %RSD for the response to solutions of 10 ppb Pb was 2.5%. Such extremely low %RSD was primarily attributed to automation of the analyzer, thereby avoiding human error and batch-to-batch variation. Other figures of merit are summarized in Table 2.

Ionic species can interfere with the voltammetric detection of Pb^+2^ if it can out-compete Pb^+2^ for the binding sites on SAMMS during the preconcentration step. From batch adsorption experiments (Yantasee et al. 2003), AcPhos–SAMMS had an affinity for metal ions in decreasing order as follows: Pb^2+^ > Cu^2+^ > Mn^2+^ > Cd^2+^ > Zn^2+^ > Co^2+^ > Ni^2+^ ~ Ca^2+^ >> Na^+^. Based on the affinity series, calcium, Zn, nickel, cobalt, and manganese could not complete with Pb for the binding sites. This corresponds to our previous article (Yantasee et al. 2005b) that these metal ions did not interfere with Pb signals even when they were present at much higher molar concentrations (e.g., 100-fold for Ca, 70-fold for Zn, Ni, Co, and 10-fold of Mn). The sensor was also robust and reliable in the SIA system, and the analyzer can be automated and remotely controlled.

## SAMMS–Graphite Ink-Modified Disposable Sensors

**Figure 7:**
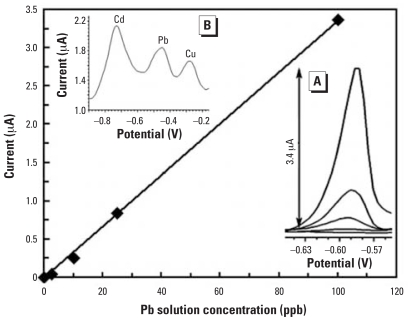
A Pb calibration curve at a 10 wt% AcPhos–SAMMS screen-printed sensor after 5-min preconcentration. Inset (*A*) shows the corresponding Pb voltammograms; inset (*B*) shows the simultaneous detection of 90 ppb Cd, 18 ppb Pb, and 18 ppb Cu. *y* = 0.034*x; R*^2^ = 0.999. Reproduced from Yantasee et al. (2005a), with permission of Elsevier.

Disposable sensors for the assay of toxic metal ions are gaining popularity because of their low costs, simplicity, and ease of use. Of all the disposable sensors, screen-printed carbon electrodes (SPCEs) coupled with stripping voltammetric techniques have been increasingly investigated. Most screen-printed electrodes for the sensitive assay of metal ions have been based on Hg film (Desmond et al. 1998; Liu et al. 1997; Wang and Baomin 1992; Wang et al. 1993b; Yang et al. 2005), or Hg oxide particles (Choi et al. 2001) as the modifier for the working electrodes. Hg-free SPEs have been developed by employing gold (Masawat et al. 2003), silver (Zen et al. 2002), bare carbon electrodes (Honeychurch et al. 2002), or by modifying electrodes with drop coating of chemical such as 1-(2-pyridy-lazo)-2-naphthol (Honeychurch et al. 2001a) or calixarene (Honeychurch et al. 2001b). However, the sensitivity, reliability, and cost competitiveness of such electrodes have yet to reach those of the Hg-based electrodes. To create Hg-free disposable sensors, we have modified the screen-printed carbon sensors with a mixture of conductive ink and 10% by weight of AcPhos–SAMMS. The screen-printed sensor (Figure 1B) consisted of three built-in electrodes on a 0.5-mm-thick plastic substrate: screen-printed carbon as working and counter electrodes, and Ag–Ag chloride electrode as the reference electrode. The screen-printed sensors were electronically connected to a hand-held potentiostat for the voltammetric analysis of Cd, Pb, and Cu as shown in Figure 7 (Yantasee et al. 2005a). Other figures of merit are presented in Table 2. Because of the strong covalent bonding and cross-linking of the functional groups on the mesoporous silica supports of SAMMS before being embedded onto the screen-printed sensors, SAMMS-based sensors can be reused for tens of measurements with minimal degradation. Being able to reuse the same screen-printed sensor many times makes establishment of the calibration curve possible on a single electrode and the costs more competitive when compared with most disposable sensors that only allow a single use.

## SAMMS–CNT Paste Electrodes

Although SAMMS has proven to be effective for the preconcentration of Pb at the electrode surface. even in complex matrices like groundwater, the high contents of proteins and macromolecules in urine, blood, and saliva still can cause the fouling of the electrodes, which results in degradation of the Pb signals. Therefore, we have reported for the first time the development of a novel sensor based on a composite of IDAA–SAMMS and CNTs in order to make a Hg-free electrochemical sensor that will virtually have no fouling when used with biological samples. The benefits of SAMMS have already been discussed, and the popularity of CNTs is growing rapidly in the field of electrochemical biosensors because of their electrocatalytic properties in the redox reactions of many important biomolecules as well as their unique property in reducing the electrode fouling caused by enzymatic products (although the antifouling mechanism is unclear), as reviewed by our research group and collaborator (Lin et al. 2005).

**Figure 8:**
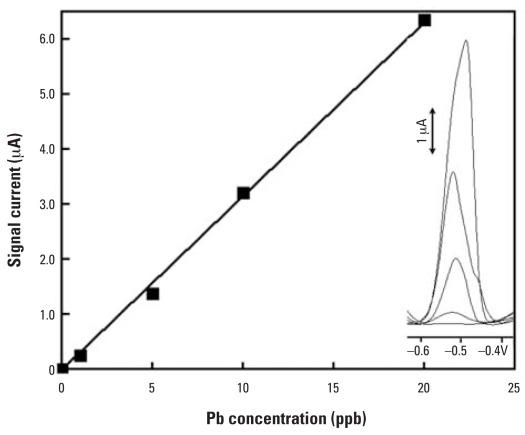
Calibration curve of Pb in samples containing 25 vol% urine measured after 5 min preconcentration at a IDAA–SAMMS–CNT paste electrode; inset shows the corresponding Pb voltammograms. *y* = 0.316*x; R*^2^ = 0.998.

At physiologic pH of urine, Pb was associated with urinary macromolecules and could not be accurately quantified. Thus, before subjecting these macromolecules to the sensor, urine specimens were prepared as summarized in Table 1. Figure 8 illustrates the linear detection range of Pb in samples containing 25 vol% urine. and the figures of merits are summarized in Table 2. The SAMMS–CNT sensors are much more sensitive than SAMMS–CPEs for detecting Pb; the Pb signals were about 9-fold larger in both deionized (DI) water and sample containing 25 vol% of rat urine. At the SAMMS–CPE, the Pb signal was virtually zero when the sample contained 25 vol% of urine after two measurements, which was most likely due to the electrode fouling. On the contrary, at the SAMMS–CNT electrode, only small signal drop occurred when the sample contained 25 vol% urine, compared with the sample containing no urine. Evidently, CNTs improved the sensitivity of Pb measurements as well as minimized the electrode fouling in rat urine. However, without IDAA–SAMMS to preconcentrate Pb, the CNT paste electrode could not detect a low parts per billion level of Pb. Thus, the composite of the two materials opens the door for a new breed of Hg-free sensors for sensitive and selective detection of toxic metal ions in biological matrices at environmentally and toxicologically relevant concentrations.

## Measurements of Background Blood Pb

The portable metal analyzer (based on FIA at Hg-film electrode) developed in this work was used successfully for measurements of background Pb in human blood obtained from a commercial supplier (Golden West Biologicals, Inc., Temecula, CA, USA). The experimental protocol was reviewed by the PNNL Human Subject Institutional Review Board and was determined to be exempt, as all samples were obtained anonymously and could not be traced to an individual. The blood samples were obtained from 10 individual adult males and subject to the portable analyzer without adding any Pb. For these samples, no information was available concerning any known environmental or occupational exposures to Pb. Sample pre-treatment procedure is summarized in Table 1. Table 4 compares Pb concentrations obtained independently by two researchers using our portable microanalyzer and the ICP-MS. The microanalyzer yielded background blood Pb levels the same as those from the state-of-the-art ICP-MS. Similar to the ICP-MS, detection of Pb by the microanalyzer was not affected by the sources (donors) of blood nor the chemicals used as anticoagulants; hence the calibration curve can be constructed in a pure medium. Without the undesirable matrix effect of blood, the microanalyzer can analyze samples from universal sources as can the ICP-MS.

## Conclusions

To successfully conduct environmental epidemiology studies, investigators need accurate and quantitative technologies for measuring xenobiotic exposure. Microanalytical based sensors that work with complex biomatrices such as blood, urine, and saliva are being developed and validated. These sensor platforms are needed to develop a personalized exposure assessment strategy that may improve our ability to make definitive associations between chemical exposures and disease.

For biomonitoring of Pb, two classes of metal analyzers have been developed at PNNL. The first class was based on flow injection analysis and stripping voltammetry of Pb at a Hg-film electrode. When used to detect Pb in blood, urine, and saliva, the analyzer has many advantages: it was very reliable, easy to use with automation, had high throughput (3 min per sample), and required only simple pretreatment of the samples that did not involve acid digestion and large sample dilution. Thus, Pb in the concentration range of relevance for its biomonitoring can be accurately quantified. In addition, being miniature in size, the analyzer had economical use of samples (e.g., ~ 60 μL per measurement), making the collection of blood less invasive (i.e., finger prick vs. blood draw), which is particularly important when sampling children. It also required less reagents (e.g., ~ 1 μg of Hg per measurement), thus minimizing the health concern of Hg use. Most important, the microanalyzer can detect Pb in urine, blood, and saliva as accurately as the ICP-MS with high reproducibility (%RSD < 5) and sensitivity (low density lipoprotein < 1 ppb), yet is much more portable, field deployable, and less expensive than the ICP-MS.

The second class of metal analyzers is made Hg-free by exploiting novel nanostructure materials; *a*) the SAMMS developed at PNNL for selectively and effectively preconcentrating Pb at the electrode surface, and *b*) the CNTs for minimizing electrode fouling and increased measurement sensitivity when used in a composite with SAMMS. Three SAMMS materials have been used in both batch and automated flow measurements of Pb as well as in disposable sensors for field screening. In addition to excellent detection sensitivity (low parts per billion level), SAMMS-based sensors have many advantages. The Pb preconcentration is short (2–5 min) and requires no electrolyte and sample degassing. Because the detection step is performed in pure acid, it has no interference and the electrode cleaning is not required. The sensors have long lives because of the stability of SAMMS. An Automated portable analyzer based on SIA/AdSV has been developed with SAMMS–carbon paste as the working electrode, which will be replaced with the SAMM–CNTs for optimal performance in biological samples.

## Figures and Tables

**Table 1:** Typical operating conditions of Pb sensors based on Hg-film electrodes and SAMMS-based electrodes.

Parameter	Condition
Flow system	FIA/ASV at Hg-film electrode
Sample preparation	*a*) Pb-spiked samples containing 10–50 vol% urine, 10 vol% bood, or 50 vol% saliva; *b*) 10-min incubation; *c*) acidified to 1 M HCl; and *d*) filtered through 5,000 NMWL cut-off ultrafilters
Sample volume	60 μL per measurement
Carrier	5 ppm Hg/0.5 M HCl
Flow rate	1 μL/sec
Preconcentration	–1.0 V, 110 sec
Detection by SWV	Scan from –0.75 V to –0.45 V
Electrode precondition	0.6 V, 90 sec in carrier
Batch measurements	SAMMS-based electrodes
SAMMS	5–10 wt% of SH-, AcPhos-, or IDAA-SAMMS
Conductive matrix	Cabon paste, graphite ink, or carbon nanotube paste
Samples	Pb in DI water, groundwater, or samples contained 25% rat urine
Sample volume	8 mL waters or 0.5 mL of urine samples
Preconcentration	2–5 min in stirred solutions at open circuit
Electrolysis	–0.8 V to –1 V for 60 sec in 0.3–0.5 M HNO_3_ or HCl
Detection by SWV	Scan from –0.7 V to –0.35 V
Electrode cleaning	Often not required

Abbreviations: AcPhos, acetamide phosphonic acid; DI, deionized; IDAA, iminodiacetic acid; SWV, square-wave stripping voltammetry.

**Table 2:** Summary of figures of merit of various analyzers for the detection of Pb.

Sensor type	Electrode specifications	Conditions	Linear slope (μA/ppb Pb)	Data range (ppb Pb)	*R*^2^	Reference
FIA/AdSV	Hg film on glassy electrode	Without urine	0.030	0–50	0.996	Yantasee et al. 2007
		With 10 vol% urine	0.032	0–10	0.953	
		With 50 vol% urine LDL = 0.44 ppb with 50% urine %RSD = 4.9 (*n* = 7, 10 ppb Pb in 50 vol% urine sample)	0.017	0–50	0.995	
		Without blood With 10 vol% blood,	0.035	0–20	0.993	Yantasee et al. 2007
		Pb spiked before protein removal	0.033	0–20	0.999	
		Pb spiked after protein removal LDL = 0.46 ppb with 10% blood %RSD = 2.4 (*n =* 10, 10 ppb Pb in 10 vol% blood sample)	0.035	0–20	0.991	
		In DI water, but similar to samples With 50 vol% saliva LDL = 1 ppb with 50% saliva %RSD = 5.0 (*n =* 7, 10 ppb Pb in 50 vol% saliva sample)	0.463^a^	0–10	0.990	Yantasee et al. 2005c
SH–SAMMS–carbon paste	5 wt% SH–SAMMS–carbon paste	Groundwater LDL = 2 ppb in groundwater %RSD = 4.8 (*n =* 5, 25 ppb Pb in groundwater)	0.025	0–50	0.997	New results
IDAA–SAMMS–CNT	10 wt% IDAA–SAMMS–CNT paste	Sample containing 25 vol% urine LDL = 1.0 ppb in sample with 25% urine %RSD = 4.5 (*n =* 6, 10 ppb Pb in DI water)	0.316	0–20	0.998	New results
SIA/AdSV	10 wt% AcPhos–SAMMS–carbon paste	DI water LDL = 1 ppb in water %RSD = 2.5 (*n =* 7, 10 ppb Pb in DI water)	0.013	0–25	0.995	Yantasee et al. 2005b
Disposable sensors	10 wt% AcPhos–SAMMS–graphite ink	DI water LDL = 0.91 ppb in water %RSD = 5 (*n=* 6, 10 ppb Pb in DI water)	0.034	0–500	0.999	Yantasee et al. 2005a

Abbreviations: DI, deionized; LDL, low density protein; NMWL, nominal molecular weight limit; nV/A, peak area, nanovolts/amp.

a Peak area was used as signal and the unit of slope was nV/A per ppb.

**Table 3:** Pb concentrations in spiked saliva, urine, and blood obtained from naïve rats.

	Pb concentration (ppb)^a^		
Biological samples	ICP-MS	Microanalyzer	Reference
Saliva	2.2 ± NA^b^	2.2 ± 0.1	Yantasee et al. 2005c
	6.2 ± 0.2	6.1 ± 0.3	
	11.6 ± 1.4^c^	11.9 ± 0.4	
Urine	13.5 ± 0.1	14.1 ± 2.0	Yantasee et al. 2007
	25.9 ± NA^b^	23.4 ± 1.2	
Blood	64.5 ± 8.0	58.0 ± 1.1	Yantasee et al. 2007
	110.9 ± 2.3	114.6 ± 0.4	

a Data are presented as mean ± SD, all measured with duplicate (*n* = 2) except

b (*n* = 1) and

c (*n* = 4).

**Table 4:** Detection of background blood Pb levels in blood obtained from 10 adult males.^a^

		Pb concentration (ppb)^b^		
Donor no.	Anticoagulant^c^	ICP-MS	Microanalyzer^d^	Percent error
1	HP	38.7 ± 0.1	36.5 ± 0.1	5.5
1	CPDA	37.9 ± 0.5	40.0 ± 1.4	5.6
1	EDTA	40.6 ± 0.0	40.4 ± 0.1	5.7
1	NaC	36.9 ± 0.1	39.0 ± 1.3	3.8
2	NaC	28.3 ± 0.7	27.2 ± 0.2	0.5
3	NaC	17.0 ± 1.0	16.6 ± 0.5	2.6
4	NaC	11.1 ± 0.4	11.6 ± 0.5	4.5
5	NaC	6.0 ± 0.0	7.8 ± 0.3	28.9
6	NaC	12.6 ± 0.5	13.1 ± 0.2	4.1
7	NaC	11.6 ± 0.6	11.7 ± 0.3	0.9
8	NaC	9.5 ± 0.3	10.0 ± 0.3	5.9
9	NaC	124.5 ± 3.8	127.8 ± 1.2	2.7
10	NaC	19.9 ± 0.5	20.6 ± 0.1	3.3

Abbreviations: CPDA, citrate, phosphate, dextrose, and adenine; EDTA, ethylenediaminetetraacetate; HP, heparin; NaC, sodium citrate.

a Operating conditions are described in Table 1.

b Results are presented as mean ± SD, all measured with duplicates.

c Blood samples were obtained from a commercial supplier of human tissues (Golden West Biologicals, Inc., CA) and were not prescreened for Pb exposure.

d Pb concentrations were calculated from Pb signals using the slope value (0.05 μA/ppb Pb) of the calibration curve constructed with 0–20 ppb of Pb in a pure medium without adding blood (see “Pb, single” curve of Figure 4).
